# 0991. Nebulized heparin reduces pulmonary inflammatory responses in a rat model of acute lung injury

**DOI:** 10.1186/2197-425X-2-S1-P76

**Published:** 2014-09-26

**Authors:** L Chimenti, R Guillamat, MN Gomez, J Tijero, T Lebouvier, L Blanch, A Artigas

**Affiliations:** Critical Care Center, Corporació Sanitária i Universitária Parc Taulì-UAB, Sabadell, Spain; Surgical Intensive Care Unit, Pontchaillou University Hospital, Rennes, France; CIBERES, Enfermedades Respiratorias, Bunyola, Spain

## Introduction

Sepsis is a mayor cause of acute lung injury (ALI). Pulmonary coagulopathy is intrinsic to ALI and in sepsis, anticoagulant system is impaired, due to consumption and downregulation by inflammatory mediators. Several studies, in ALI patients and in ALI experimental models, inconsistently suggest beneficial effects of systemic anticoagulants which affect systemic coagulation. Nebulization of anticoagulants might allow for higher pulmonary concentration and reduce the risk of systemic bleeding.

## Objectives

To assess the effects of local heparin treatment in a rat model of acute lung injury.

## Methods

Adult male Sprague-Dawley rats (250-300 g; n=6/group) were anesthetized with isofluorane and subjected to intratracheal administration (IA) of LPS (10 µg/g b.w.). Saline or heparin (1000 IU/kg) were nebulized at 4 and 8h after LPS instillation. Animals were sacrificed 24h after the injury. Inflammatory cells and total proteins were assessed in bronchoalveaolar lavage fluid (BALF). IL-6, GRO-κC, TNF-α and IL-10 were measured in lung homogenate by multiplex assay (Luminex, Merck Millipore, Darmstadt, Germany). Data are reported as mean±SD. One-way ANOVA was used for multigroup comparisons.

## Results

In BALF, nebulized heparin significantly reduced neutrophils in animals instilled with LPS (12.4±4.3x107 cells/ml) compared to animals administrated with LPS and nebulized with saline (18.1±6.1x107 cells/ml, p< 0.05). Total BALF proteins were found to be lower in rats nebulized with heparin (578.3±89.8 µg/ml) than in rats treated with saline (914.6±250.1 µg/ml, p< 0.005). In lung homogenate, IL-6, TNF-α, GRO-κC levels significantly decreased in animals nebulized with heparin compared to those ones nebulized with saline (IL6: LPS+Sal: 147.7±1.3 ng/ml, LPS+Hep: 47.3±20.2 ng/ml, p< 0.005; TNF-α: LPS+Sal: 1.1±0.2 ng/ml, LPS+Hep: 0.3±0.1 ng/ml, p< 0.005; GRO-κC: LPS+Sal: 45.9±22.9 ng/ml, LPS+Hep: 40.9±9.8 ng/ml, p< 0.005). IL-10 did not show any significant difference among groups.

## Conclusions

Our results show that local heparin administration reduces pulmonary inflammatory responses in a rat model of acute lung injury.

